# Tranilast inhibits angiotensin II-induced myocardial fibrosis through S100A11/ transforming growth factor-β (TGF-β1)/Smad axis

**DOI:** 10.1080/21655979.2021.1982322

**Published:** 2021-10-18

**Authors:** Youquan Chen, Ming Huang, Yi Yan, Dequan He

**Affiliations:** Department of Cardiology, Third Affiliated Hospital of Guangzhou Medical University, Guangdong, China

**Keywords:** Tranilast, myocardial fibrosis, S100A11, TGF-Β1/Smad axis

## Abstract

Tranilast has an ameliorative effect on myocardial fibrosis (MF), but the specific mechanism has not been studied. S100A11 is a key regulator of collagen expression in MF. In this paper, we will study the regulatory roles of Tranilast and S100A11 in MF. After the introduction of angiotensin II (AngII) to Human cardiac fibroblasts (HCF), Tranilast was administered. CCK-8 kit was used to detect cell viability. Wound Healing assay detected cell migration, and Western blot was used to detect the expression of migration-related proteins and proteins related to extracellular matrix synthesis. The expression of α-SMA was detected by immunofluorescence (IF). The expression of S100A11 was detected by qPCR and Western blot, and then S100A11 was overexpressed by cell transfection technology, so as to explore the mechanism by which Tranilast regulated MF. In addition, the expression of TGF-β1/Smad pathway related proteins was detected by Western blot. Tranilast inhibited Ang II–induced over-proliferation, migration and fibrosis of human cardiac fibroblasts (HCF), and simultaneously significantly decreased S100A11 expression was observed. Overexpression of S100A11 reversed the inhibition of Tranilast on AngII–induced over-proliferation, migration, and fibrosis in HCF, accompanied by activation of the TGF-β1/Smad pathway. Overall, Tranilast inhibits angiotensin II-induced myocardial fibrosis through S100A11/TGF-β1/Smad axis.

## Introduction

Myocardial fibrosis (MF) is a common pathological manifestation in the late stages of plenty of cardiovascular diseases whose main pathological features, including the proliferation of cardiac fibroblasts and abnormal accumulation of collagen, lead to adverse pathophysiological events like ventricular remodeling, myocardial dysfunction and even heart failure [1,2]. MF is closely related to the occurrence and development of hypertension, hypertrophic cardiomyopathy and heart failure [3,4]. At present, the lack of specific treatment for MF calls for the identification of a treatment method for MF management.

Originally discovered and used in the treatment of allergic diseases, tranilast is a drug that inhibits the release of bioactive substances from mast cells (MC) and basophil degranulation [5]. In recent years, it has been found to have anti-fibrotic, anti-inflammatory and anti-oxidative stress effects [6–8]. Study showed that Tranilast alleviates MF in diabetic rats through the TGF-β/Smad pathway [9]. Early and delayed Tranilast therapy can reduce pathologic fibrosis after myocardial infarction [10]. Tranilast can alleviate MF by inhibiting monocyte/macrophage infiltration in DOCA/ salt-induced hypertension rats [11]. However, there has existed no clear report on the mechanism of the inhibitory effect of Tranilast on MF.

As a member of the S100 protein family, S100A11 is mainly expressed in vascular endothelial cells or smooth muscle cells and is involved in a variety of intracellular activity regulation [12,13]. Differentiated proteomics revealed that S100A11 was a key factor for the collagen expression of human cardiac fibroblasts induced by aldosterone, and CRISPR/Cas9-mediated S100A11 gene knockout blocked the collagen production of human cardiac fibroblasts induced by aldosterone [14]. Downregulation of S100A11 in vivo significantly inhibited the development of inflammation and liver fibrosis in choline/methionine-deficient mice [15]. In addition, Tranilast can block the interaction between S100A11 and the advanced glycation end product receptor (RAGE) V domain, thereby inhibiting cell proliferation [16].

Therefore, we hypothesized that Tranilast plays an important role in Ang-II induced fibrosis by regulating S100A11 and in this paper, we tested whether Tranilast can play a regulatory role in MF by regulating S100A11 and its downstream signaling molecules. Our study provides a solid experimental basis for the clinical treatment of MF by Tranilast.

## Materials and methods

### Cell culture and treatment

Human Cardiac fibroblasts (HCF) were purchased from the Shanghai FuHeng Biology (Shanghai, China) and cultured in DMEM medium (Gibco; Thermo Fisher Scientific) supplemented with 10% FBS (Gibco; Thermo Fisher Scientific) at 37°C, and 5% CO_2_ in a humidified atmosphere incubator. When the cells grew to 50%-60%, the cells were pretreated with Tranilast (1 μM, 3 μM, 10 μM and 30 μM) for 30 min and then treated with 100 nM AngII for 48 h. The cells were grouped into Control, Ang II, Ang II + 1 μM, Ang II + 3 μM, Ang II + 10 μM, Ang II + 30 μM.

### CCK-8

Cells (5 × 10^3^) were plated in 96-well plates and cell growth was determined by using CCK8 assay. After the cells were pretreated with Tranilast (1 μM, 3 μM, 10 μM and 30 μM) for 30 min and then treated with 100 nM AngII for 48 h, cells were given 10 μL CCK-8 solution and cultured for 3 h. Finally, the OD value was detected at the wavelength of 450 nm [17].

### Wound healing

Cells were cultured in 6‐well plates and allowed to grow to ~95% confluence. The linear wound was created by a plastic pipette tip. The linear wound was then photographed under a microscope at 0 or 24 h.

### Western blot

Western blot analysis was performed under conventional conditions [18]. Protein samples were prepared using lysis buffer, and then loaded and electrophoresed with the running gel. Transfer buffer was used for protein transferring, and the membrane was blocked with 5% skim milk followed by primary and secondary antibody staining. Protein blots were visualized with the method of chemiluminescence. The antibodies used were rabbit anti- MMP2 antibody (1:1000, 40994S, CST, USA), rabbit anti- MMP9 antibody (1:1000, 13667S, CST, USA), rabbit anti- Collagen I antibody (1:1000, 72026S, CST, USA), rabbit anti- Collagen III antibody (1:1000, 66887S, CST, USA), rabbit anti-α-SMA antibody (1:1000, 19245S, CST, USA), rabbit anti- Fibronectin antibody (1:1000, 26836S, CST, USA), rabbit anti- connective tissue growth factor (CTGF) antibody (1:1000, 86641S, CST, USA), rabbit anti- connective tissue growth factor (CTGF) antibody (1:1000, 86641S, CST, USA), rabbit anti- connective tissue growth factor (CTGF) antibody (1:1000, 86641S, CST, USA), rabbit anti-S100A11 antibody (1:1000, ab180593, Abcam, USA), rabbit anti- TGF-β antibody (1:1000, ab215715, Abcam, USA), rabbit anti- p-Smad 2 antibody (1:1000, 18338S, CST, USA), rabbit anti- p-Smad 3 antibody (1:1000, 9520S, CST, USA), rabbit anti- Smad2 antibody (1:1000, 5339S, CST, USA), rabbit anti-Smad 3 antibody (1:1000, 9523S, CST, USA) and rabbit anti-GAPDH antibody (1:1000, 5174S, CST, USA). Goat Anti-Rabbit IgG H&L (Alexa Fluor® 488) (ab150077, Abcam, Cambridge, MA, USA) was used as the secondary antibody.

### Immunofluorescence (IF)

The cells were fixed with 4% paraformaldehyde for 10 min and then transfused with 0.2% TritonX-100 (order number: A600198; Sangon Biotech, Shanghai, China) at room temperature for 5 min. Subsequently, 5% bovine protein serum (Gibco; Thermo Fisher Scientific) was incubated for 1 h for sealing. It was then incubated overnight with primary antibody α-SMA (1:200, ab5831, Abcam) at 4°C. The corresponding secondary antibody (1:500, ab150113, Abcam) was then placed at room temperature under dark conditions for 1 h and stained with Hoechst 33,342 (1:1000 PBS) at room temperature for 3 min. Images were captured using a Nikon A1MP+ confocal microscope system (Nikon, Tokyo, Japan).

### RT-qPCR

Based on the manufacturer’s instructions, we isolated the total RNA from cells using Trizol reagent (Life Technologies, Carlsbad, CA, USA, the single-stranded cDNAs were synthesized from 1 μg of RNA. The expression of mRNAs was quantified by RT-PCR with SYBR Green I (Thermo Fisher Scientific, Inc). Additionally, PCR primers used for amplification of S100A11 are 5′- CCGCATGATGAAGAAACTGG-3′ (S100A11 sense) and 5′- TGCATGAGGTGGTTAGTGTG-3′ (S100A11 antisense). GAPDH was used as an internal reference with 5′- GGTGGTCTCCTCTGACTTCAACA-3′ (GAPDH sense) and 5′-GTTGCTGTAGCCAAATTCGTTGT-3′ (GAPDH antisense). The quantification was performed using the 2^−ΔΔCt^ method .

### Cell transfection

The S100A11 overexpression plasmid (Oe-S100A11) was obtained from FitGene (FitGene BioTechnology CO., LTD, Guangzhou, China) using the trans-ov vector. The Oe-S100A11 plasmid and control vector (negative control, NC; 1 μg each) were transfected into cells using Lipofectamine® 2000 (Invitrogen; Thermo Fisher Scientific, Inc.), according to the manufacturer’s instructions. Following incubation for 48 h, cells were used for subsequent experiments.

### Statistical analysis

SPSS20.0 was used to analyze the data, which were expressed as means ± standard of mean (*SD*). One-way ANOVA was employed to assess differences among different groups, followed by Tukey’s post-hoc test. GraphPad Prism 5.0 software was adopted to dispose all the photographs. *P* < 0.05 was considered as statistically significant. Each experiment was repeated at least three times.

## Results

### Tranilast inhibited AngII-induced overproliferation and migration of HCF cells

**Figure 1.**
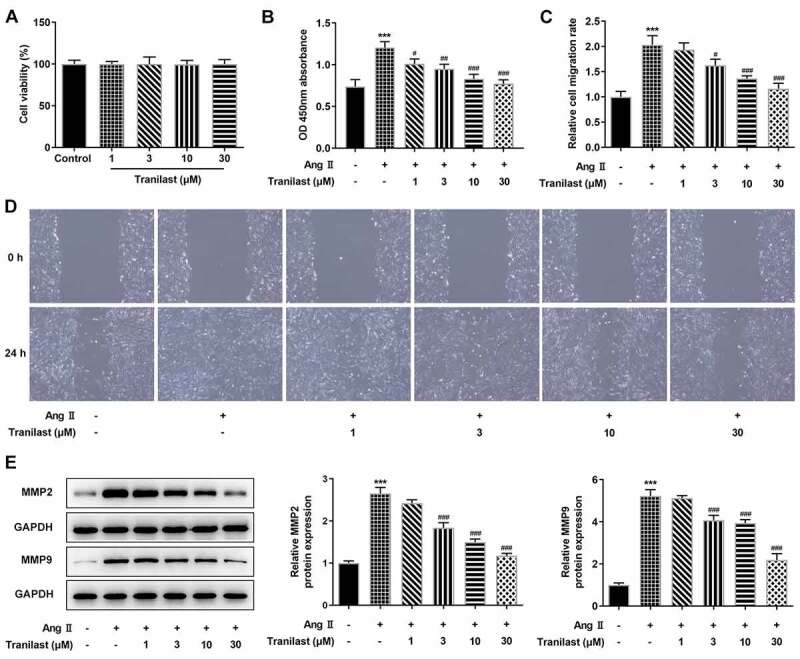
Tranilast inhibited AngII-induced overproliferation and migration of HCF cells. A. CCK-8 assay detected the cell viability after induction with different concentrations of Tranilast. B. CCK-8 assay detected the cell viability of AngII-induced cells after induction with different concentrations of Tranilast. C and D. wound healing detected the migration of cells. E. Western blot detected the expression of MMP2 and MMP9. *P < 0.05, **P < 0.01, ***P < 0.001 vs control; #P < 0.05, ##P < 0.01, ###P < 0.001 vs Ang II

Tranilast at different concentrations (1 μM, 3 μM, 10 μM and 30 μM) was administered in HCF cells and cell viability was detected using CCK-8. We found no cytotoxicity of Tranilast in this concentration range (Figure 1a). Subsequently, HCF cells were induced by Ang II and Tranilast was administered. We found that cell viability increased significantly after administration of AngII, while cell viability decreased in a dose-dependent manner of Tranilast (Figure 1b). Wound Healing results showed that compared with the control group, cell migration in the AngII group was significantly increased,; while compared with the AngII group, cell migration in the AngII+Tranilast with different concentrations decreased in a dose-dependent manner (Figure 1c and d). Western blot analysis on the expression of MMP2 and MMP9 showed that the expression of MMP2 and MMP9 was significantly increased after administration of AngII, while Tranilast inhibited the effect of AngII on MMP2 and MMP9 expression (Figure 1e). These results indicated that Tranilast inhibited the overproliferation and migration of HCF cells induced by AngII.

### Tranilast inhibited AngII-induced fibrosis of HCF cells

**Figure 2.**
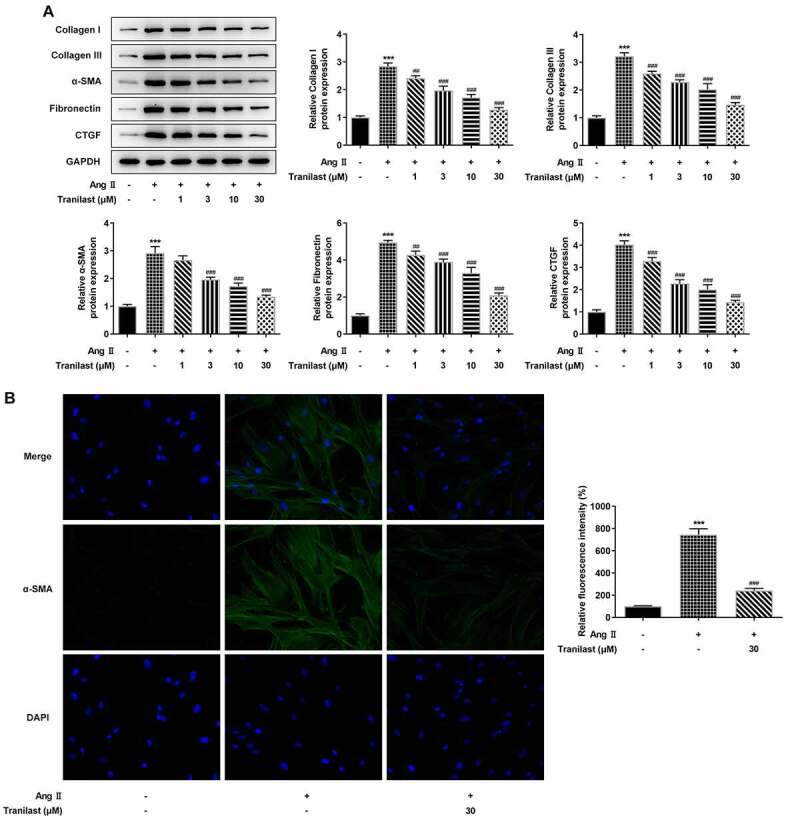
Tranilast inhibited AngII-induced fibrosis of HCF cells. A. The expression of proteins related to ECM synthesis was detected by Western blot. B. The expression of α-SMA was detected by IF. ***P < 0.001 vs control; ##P < 0.01, ###P < 0.001 vs Ang II

In order to detect the degree of cellular fibrosis, Western blot was used to detect the expressions of proteins including Collagen I, Collagen III, α-SMA, Fibronectin and connective tissue growth factor (CTGF). We found that compared with the control group, the expressions of Collagen I, Collagen III, α-SMA, Fibronectin and CTGF in the AngII group were significantly increased. Compared with the AngII group, the protein expression in AngII + 1 μM, AngII+3 μM, AngII+10 μM, and AngII+30 μM groups decreased significantly, and the decrease trend was most obvious in the AngII + 30 μm group (Figure 2a). Therefore, we detected the expression of α-SMA in cells treated with 30 μM Tranilast. The results showed that the expression of α-SMA in the AngII group was significantly increased compared with the control group, and the expression of α-SMA in the AngII + Tranilast (30 μm) group was inhibited compared with the AngII group (Figure 2b). These results indicated that Tranilast inhibited AngII–induced fibrosis of HCF cells.

### Overexpression of S100A11 reversed the inhibitory effect of Tranilast on AngII-induced overproliferation, migration and fibrosis of HCF cells

**Figure 3.**
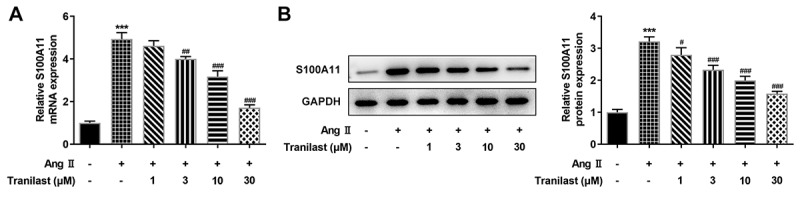
Tranilast down-regulated the expression of S100A11. A. RT-qPCR detected the expression of S100A11. B. Western blot detected the expression of S100A11. ***P < 0.001 vs control; #P < 0.05, ##P < 0.01, ###P < 0.001 vs Ang II

During the experiment, we found that the expression of S100A11 was abnormal. RT-qPCR and Western blot results showed that the expression of S100A11 increased after AngII induction compared with the control group. The expression of S100A11 decreased in a dose-dependent manner after administration of tranilast compared with the AngII group (Figure 3a and b). These results indicated that Tranilast could down-regulate the expression of S100A11.

**Figure 4.**
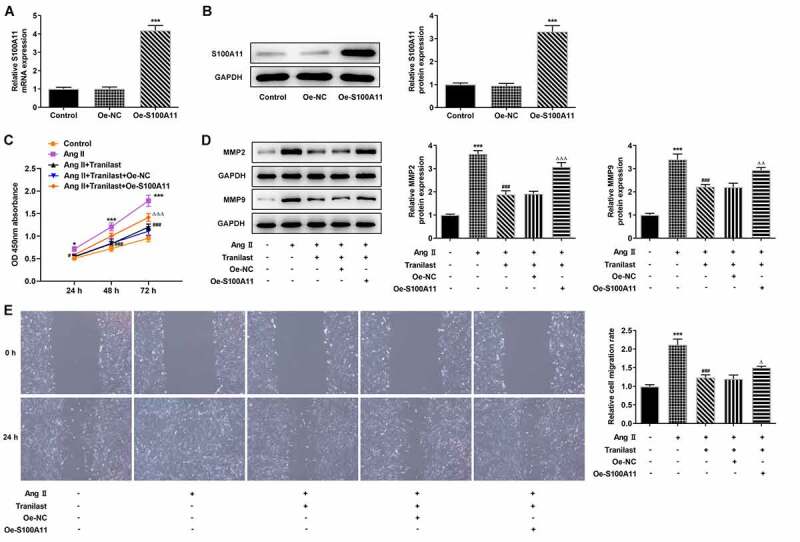
Overexpression of S100A11 reversed the inhibitory effect of Tranilast on AngII-induced overproliferation and migration of HCF cells. A. RT-qPCR detected the expression of S100A11 after cell transfection. B. Western blot detected the expression of S100A11 after cell transfection. ***P < 0.001 vs Oe-NC. C. CCK-8 assay detected the cell viability of AngII-induced cells after induction with different concentrations of Tranilast and cell transfection. D. Western blot detected the expression of MMP2 and MMP9. E. wound healing detected the migration of cells. ***P < 0.001 vs control; ###P < 0.001 vs Ang II; ΔP<0.05, ΔΔP<0.01, ΔΔΔP<0.001 vs Ang II + Tranilast + Oe-NC

**Figure 5.**
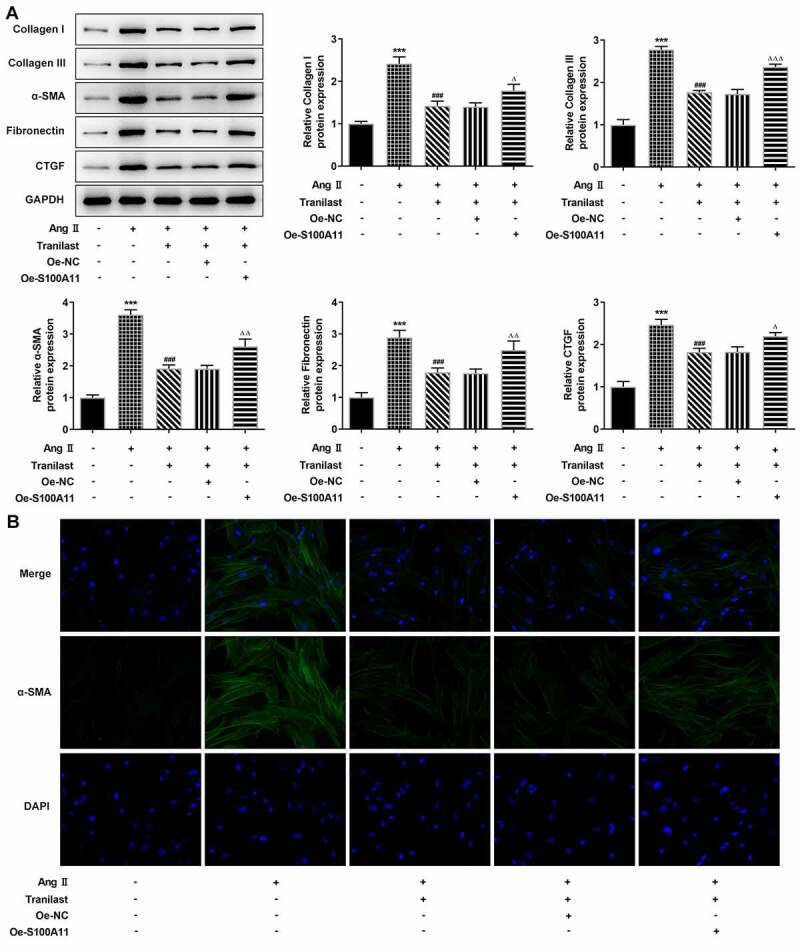
Overexpression of S100A11 reversed the inhibitory effect of Tranilast on AngII-induced fibrosis of HCF cells. A. The expression of proteins related to ECM synthesis was detected by Western blot. B. The expression of α-SMA was detected by IF. ***P < 0.001 vs control; ###P < 0.001 vs Ang II; ΔP<0.05, ΔΔP<0.01, ΔΔΔP<0.001 vs Ang II + Tranilast + Oe-NC

Therefore, we constructed the overexpressed plasmid of S100A11, and the results of RT-qPCR and Western blot showed that the cells were successfully transfected (Figure 4a and b). In addition, we selected Tranilast at the concentration of 30 μM for subsequent experiments, and divided the cells into control, AngII, AngII+ Tranilast, AngII+ Tranilast + Oe-NC and AngII+ Tranilast + Oe-S100A11 groups. CCK8 kit showed a significant increase in cell viability in the Ang II +Tranilast+Oe-S100A11 group compared to the Ang II+Tranilast+Oe-NC group (Figure 4c). Subsequently, the cell migration ability was detected, and the results showed that compared with the Ang II+Tranilast + OE-NC group, the cell migration ability in the Ang II + Tranilast + OE-S100A11 group was significantly increased (Figure 4e).This was accompanied by increased expression of MMP2 and MMP9 proteins (Figure 4d).Western blot results showed that compared with Ang II+Tranilast+Oe-NC group, the Collagen I, Collagen III, α-SMA, Fibronectin and CTGF in Ang II+Tranilast+Oe-S100A11 group were significantly increased (Figure 5a). The results of IF showed that overexpression of S100A11 reversed the inhibition of α-SMA by Tranilast (Figure 5b). The above experimental results showed that overexpression of S100A11 reversed the inhibitory effect of Tranilast on the excessive proliferation, migration and fibrosis of AngII–induced HCF cells.

### Tranilast regulated the TGF-β1/Smad pathway

**Figure 6.**
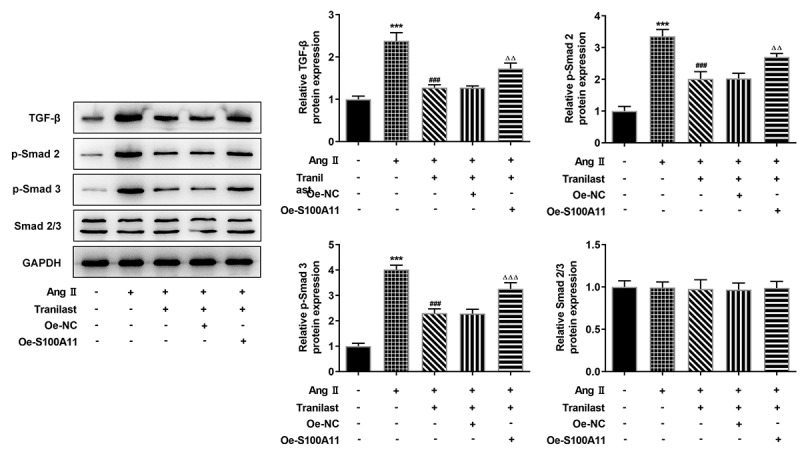
Tranilast regulated the TGF-β1/Smad pathway. The expression of proteins related to TGF-β1/Smad pathway was detected by Western blot. ***P < 0.001 vs control; ###P < 0.001 vs Ang II; ΔΔP<0.01, ΔΔΔP<0.001 vs Ang II + Tranilast + Oe-NC

Western blot was used to detect the expression of TGF-β1/Smad pathway. Compared with the control group, TGF-β, p-Smad 2 and p-Smad 3 expressions were increased in the AngII group. Compared with AngII group, TGF-β, p-Smad 2 and p-Smad 3 expressions were inhibited in AngII + Tranilast group. Compared with the Ang II + Tranilast + Oe-NC group, the expressions of TGF-β, p-Smad 2 and p-Smad 3 proteins were significantly increased in the Ang II + Tranilast + Oe-S100A11 group (Figure 6). Therefore, we preliminarily concluded that overexpression of S100A11 reversed Tranilast’s inhibition of AngII–induced excessive proliferation, migration and fibrosis in HCF cells, and this process may be achieved through the regulation of TGF-β1/Smad pathway.

## Discussion

AngII is a kind of cytokine with many biological functions, including the growth factor-like effect. By mainly binding to its receptor AT1, AngII can promote the occurrence of MF as it can not only induce cardiomyocyte hypertrophy, but also stimulate the proliferation of cardiac fibroblasts and collagen synthesis, leading to myocardial fibrosis [19,20]. AngII plays an important role in the development of myocardial fibrosis [21]. Therefore, in this paper, we used 100 nM AngII to induce HCF cells for the simulation of fibrosis for 48 h. The significant increases in fibrosis-related indicators after Ang II induction indicated the occurrence of fibrosis.

Tranilast is a fattening cell stabilizer, mainly used to treat allergic reactions [22]. Current studies have shown that Tranilast inhibits fibrosis in multiple organs, such as renal fibrosis, liver fibrosis, and myocardial fibrosis. Tranilast prevents renal interstitial fibrosis by blocking mast cell infiltration in a rat model of diabetic nephropathy [23]. Tranilast has a significant beneficial effect on the progression of liver fibrosis in praziquantel treated or untreated mice infected with Streptococcus mansoni [24]. Tranilast reduces pathologic fibrosis after myocardial infarction [10]. In our experiment, it was also found that Tranilast inhibited AngII-induced HCF cell viability, cell migration, ECM synthesis and cell fibrosis.

During the experiment, we found that the expression of S100A11 was significantly increased in AngII-induced fibrosis cells, while the expression of S100A11 was significantly decreased in cells induced by Tranilast. Through literature review, it was found that the Tranilast blocked the interaction of S100A11 with the advanced glycation end product receptor (RAGE) V domain and inhibited cell proliferation [16]. Therefore, in order to further explore the mechanism of the inhibition of cell fibrosis by Tranilast, we overexpressed S100A11 by cell transfection technology, finding that the overexpression of S100A11 could reverse the inhibition of excessive proliferation, migration and fibrosis of AngII-induced HCF cells by Tranilast. Therefore, we concluded that Tranilast inhibited Ang II-induced myocardial fibrosis by down-regulating S100A11.

Under normal conditions, the structural integrity and normal function of the heart are maintained thanks to the relatively static growth and secretion of an appropriate amount of collagen and other ECM by cardiac fibroblasts [25]. Upon activation by diseases such as myocardial infarction, cardiac fibroblasts will proliferate and differentiate into myofibroblasts with high expression of α-smooth Muscle Actin (α-SMA), resulting in excessive deposition of ECM such as Collagen I and Collagen III in fibrotic myocardium [26,27]. In addition, CTGF can stimulate the increase of ECM synthesis and the secretion of collagen by myocardial fibroblasts, leading to myocardial fibrosis [28]. Therefore, in order to detect the degree of cell fibrosis, we detected the expression of α-SMA, Collagen I, Collagen III, α-SMA, CTGF and Fibronectin protein secreted by HCF cells in our paper.

Study has shown that S100A11 promotes epithelial mesenchymal transformation of intrahepatic cholangiocarcinoma induced by TGF-β1 through the Smad2/3 signaling pathway [29]. S100A11 promotes invasion of colorectal cancer by regulating TGFβ/Smad signaling [30]. In addition, Tranilast alleviates myocardial fibrosis in diabetic rats through the TGF-β/Smad pathway [9]. TGF-β1 dominates the hardness of ECM and induces differentiation of human cardiac fibroblasts into myofibroblasts [31]. Therefore, we hypothesized that Tranilast could inhibit Ang II-induced myocardial fibrosis by down-regulating S100A11/TGF-β1/Smad axis. And our experiments have initially confirmed what we suspected. Of course, in the subsequent experiments, we will further verify our experimental results by adding pathway inhibitors or agonists.

Our paper also has some limitations. For example, our paper was only verified in cell experiments, but not in animal experiments. We will further verify in animal experiments in the following experiments. In addition, it has been reported that the effect of S100A11 is mediated by binding the receptor RAGE on its cytoplasmic membrane [16]. Therefore, it may be that S100A11 regulates TGF-β1/Smad Axis by binding to receptor RAGE. We will further explore the mechanism in the following experiments.

## Conclusion

In conclusion, our paper confirmed that Tranilast inhibited Ang II-induced myocardial fibrosis through S100A11/TGF-β1/ Smad axis. Our paper provided a solid theoretical basis for the treatment of cardiac fibrosis by Tranilast.

## Data Availability

The datasets analyzed during the current study are available from the corresponding author on reasonable request.

## Highlights

Tranilast inhibits Ang II - induced myocardial fibrosis

Tranilast down-regulates S100A11/TGF-β1/Smad Axis

S100A11 is up-regulated in Ang II - induced myocardial fibrosis
